# The effect of green carbon Dots/Titanium dioxide for photocatalytic reduction of CO_2_

**DOI:** 10.1038/s41598-026-63896-z

**Published:** 2026-08-03

**Authors:** Ahmed M. Enew, Nora Yehia Selem, N. F. El Hussieny, Samah A. Hawash, El-Shimaa M. El-Zahed

**Affiliations:** 1 Department of Chemical Engineering, Menoufia Higher Institute of Engineering and Technology (MNF-HIET), Menoufia, Egypt; 2 Department of Chemical Engineering, Higher Technological Institute (HTI), 10th, Ramadan City, Egypt; 3 Department of Basic Science, Faculty of Engineering, City University of Cairo, Cairo, Egypt

**Keywords:** Nano titanium oxide, Carbon dots, Photocatalytic reduction, Reduction of CO_2_, Chemistry, Environmental sciences, Materials science, Nanoscience and technology

## Abstract

With the rapid rate of industrial modernization, the continuous emissions of carbon dioxide (CO_2_) are greatly disturbing the natural carbon balance and contributing to global warming. To mitigate this effect, photocatalytic reduction of carbon dioxide has gained popularity as a means of converting it into useful products. This study examined the photocatalytic reduction of aqueous CO_2_ under ultraviolet irradiation (UV) using green carbon dots (CDs) made from pomegranate peel, nano titanium dioxide (TiO_2_), TiO_2_/CDs composite, and metal-loaded carbon dots-modified titanium oxide (Cu-CDs/TiO_2_). The reduction of CO_2_ gas in water was carried out in a batch reactor utilizing the four photocatalysts. In order to verify the catalysts’ functional groups, nanoscale shape, and composite production, FTIR and TEM were used for characterization. The findings demonstrated that oxygenated products were preferred by pure CDs, but the performance of TiO_2_ was very pH dependent, with ester formation being promoted in acidic environments and CO formation in alkaline ones. An increase in CO selectivity resulted from the TiO_2_-CDs composite’s enhanced charge separation and electron transfer; a change toward decreased hydrocarbons in product distribution was brought about by the addition of Cu, which opened hydrogenation pathways.

## Introduction

Photocatalytic reduction of CO₂ is a promising approach to address both environmental and energy challenges by converting a major greenhouse gas into valuable fuels and chemicals using UV light. This process harnesses the power of photocatalyst materials that can absorb light and induce chemical reactions. Among the most exciting developments in this field are the use of green conductors such as carbon dots (CDs) and nanomaterial catalysts like titanium dioxide (TiO₂).

CDs are prepared from the hydrothermal decomposition of pomegranate. It is a class of zero-dimensional carbon-based nanomaterials that exhibit remarkable optical and electronic properties, including high photostability, tunable photoluminescence, and excellent biocompatibility, making them suitable for environmental applications^1^. On the other hand, TiO₂ is a well-known photocatalyst due to its strong oxidative power, stability, and low cost. However, its efficiency is often limited by its wide bandgap, which restricts absorption to the UV region of the spectrum^2–4^. A TiO_2_/CDs hybrid system was synthesized via hydrothermal treatment. The integrated CDs with TiO₂ can enhance photocatalytic performance by expanding light absorption, facilitating charge separation, and suppressing electron-hole recombination. Consequently, this provides a more efficient route for CO₂ reduction under ultraviolet light. This synergistic approach maximizes solar energy utilization while advancing sustainable, eco-friendly technologies for carbon capture and conversion^5,6^. Furthermore, the CDs/TiO_2_ were dropped with Cu(II) to elevate its impact on the production efficiency of methanol (CH_3_OH) and carbon monoxide (CO) production.

Various studies have suggested mixing TiO_2_ with metal, nitrogen, and carbon to enhance the photocatalytic efficiency of the catalyst^7–9^. Haoran Liu et al., in 2024^10^, documented a photoredox system for the reduction of CO_2_, alongside the oxidation of biomass-derived amines. Double-shelled CdS nanocages (CdS DSNC) are fabricated by a progressive etching sulfuration method, yielding remarkable CO and difurfurylamine outputs of 1226.4 and 5526.5 µmol·g^− 1^·h^− 1^, respectively. Mechanistic investigations demonstrate that the double-shelled architecture provides CdS DSNC with enhanced accessibility to active sites and a reduced transfer distance for photocarriers. Moreover, furfurylamine functions as both an electron donor and a CO_2_ sequester, thus enhancing photoredox efficacy.

Changhai Liu et al. in 2023^11^ Novel CDs modified cadmium sulfide (CdS) hollow spheres (CDs/CdS) were synthesized by a multistep procedure involving template assembly, template removal, and ultrasonic impregnation. This heterostructure, devoid of noble metals, enhances solar-driven CO_2_ reduction efficiency, achieving an ideal CDs/CdS yield rate of 16.09 µmol g^− 1^ h^− 1^, approximately quadrupling the performance of a pristine CdS hollow sphere under identical conditions. CDs/CdS demonstrate superior performance and stability owing to the formation of hollow spheres and seamless contact, which provide substantial interior volume, close interfacial contact for charge transport, and enhanced concentration of sacrificial agents. Concurrently, as cocatalysts, CDs enhance charge separation and stabilize electrons.

This study focused on synthesizing a carbon dot (CD) photocatalyst and combining it with TiO_2_ and metal-loaded CDs to create three photocatalysts: CDs, CDs/TiO_2_, and Cu-CDs/TiO_2_, and investigated the photocatalytic reduction of aqueous CO_2_ to useful products under ultraviolet light.

## Materials and methods

### Materials

The material specifications and suppliers are given in Table 1. All these chemicals were used and were of analytical grade. Also, distilled water was used throughout the entire experiment.

**Table 1:** Membrane raw materials specifications and suppliers.

Materials	Specification	Supplier
Hydrochloric Acid (HCL)	MW: 36.5 Assay%: 30	El-Gomhouria Co
Sodium hydroxide (NaOH)	MW:40 Assay%: 97	Sigma Aldrich
Titanium dioxide (TiO_2_)	Density: 4260 kg. m^− 3^ MW: 80	Sigma Aldrich
Ethanol (C_2_H_5_OH)	Density: 0.791 kg. m^− 3^ MW: 46.07 Assay%: 99.8	Sigma Aldrich

## Methods

### Preparation of nano carbon dots

**Fig. 1:**
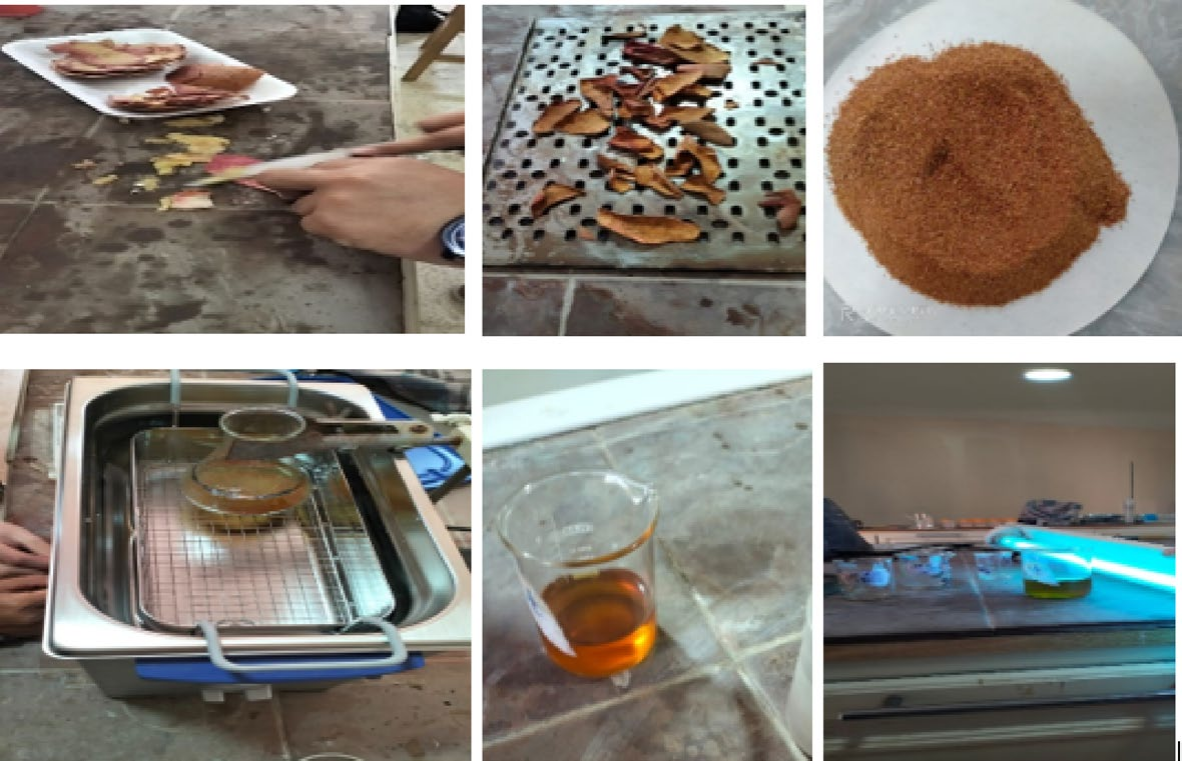
Preparation steps of CDs.

CDs were synthesized from pomegranate peel. The peel was washed with distilled water, dried in an oven at 70 ℃, and then crushed into a fine powder, as illustrated in Fig. 1. Weigh 0.3 g of fine peel powder and mix it with 45 mL of purified water. The solution underwent sonication for 60 min before being transferred to a 750 mL Teflon-lined stainless steel autoclave. Position the autoclave in the oven at 100 ℃ for a duration of 10 h. Subsequent to cooling, the solution underwent filtration twice using a 22 μm filter membrane to eliminate substantial insoluble carbonaceous particulates. The CDs were harvested after 48 h of dialysis using a dialyzing membrane to eliminate contaminants. The resultant supernatant was preserved at 4 ℃ for subsequent utilization^12^.

### Preparation of nano titanium oxide

**Fig. 2:**
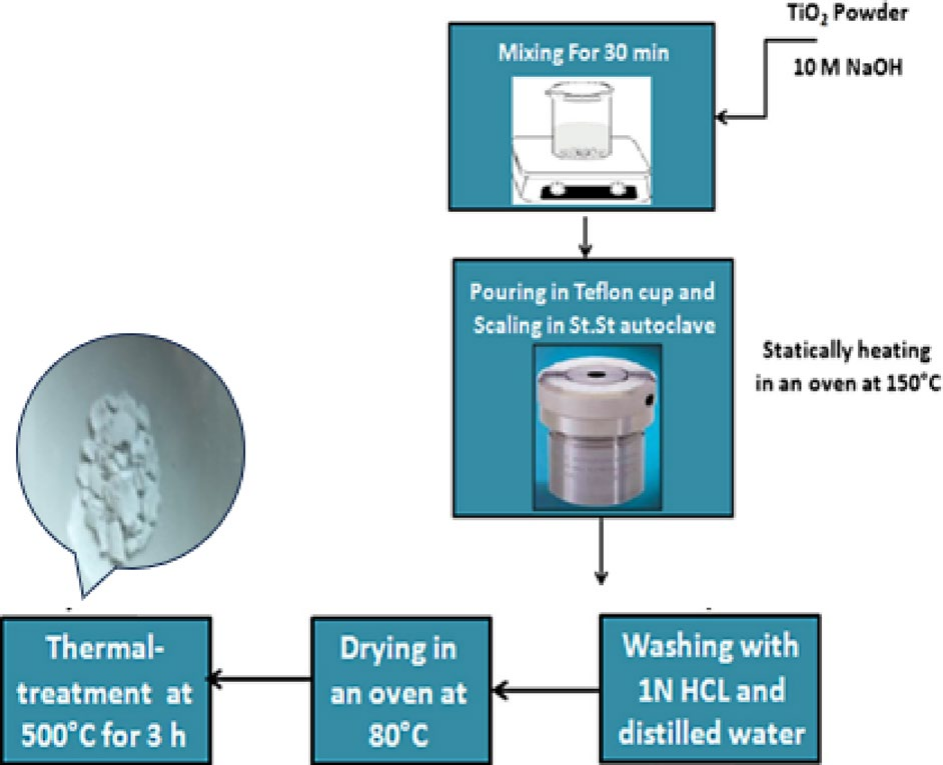
Hydrothermal synthesis method of nano TiO_2_.

Nano TiO_2_ was prepared using a hydrothermal process. 4 g of commercial TiO_2_ powder was mixed with 400 mL of 10 M NaOH_(aq)_. The mixture was stirred at room temperature for 30 min, then heated in a 1 L Teflon-lined autoclave at 150 °C. White powder precipitate was filtered and washed with 1 N HCl_(aq)_ and deionized water. Dried at 80 °C in an oven for 4 h and calcinated in a muffle at 500 °C for 3 h to yield nano TiO_2_. Figure 2 shows a schematic diagram of the hydrothermal synthesis of nano TiO_2_^13^.

### Preparation of nano TiO_2_ /CDs composite

**Fig. 3:**
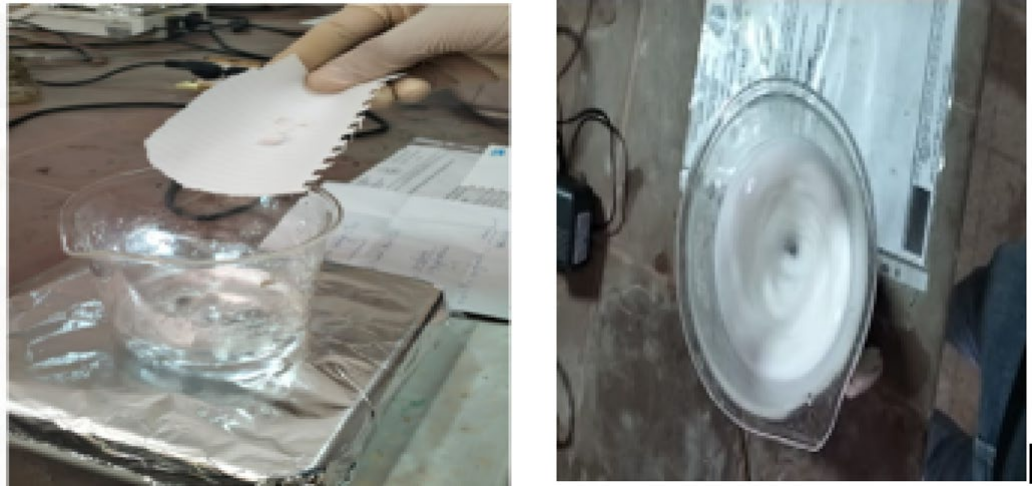
Preparation of nanoTiO_2_ /CDs composite.

Mix 0.05 g of TiO_2_ nanopowder and 50 g of C_2_H_5_OH, and ultrasonically disperse for 15 min. Heat and stir the mixture at 55–60 °C. Gradually add 0.5 mL of deionized water and 0.5 mL of Tetraethyl orthosilicate (TEOS) dropwise. Mix the mixture for 6 h with stirring, and centrifuge at 10,000 rpm for 8 min. Supernatant powder was obtained from the solution and washed three times with an ethanol solution, as shown in Fig. 3. The powder was dissolved in 100 mL of distilled water, followed by 0.5 mL of CDs aqueous solution (0.1 mg/L), and the mixture was magnetically stirred at room temperature for 6–8 h to completely combine CDs with TiO_2_^14^.

### Preparation of the metal composite

**Fig. 4:**
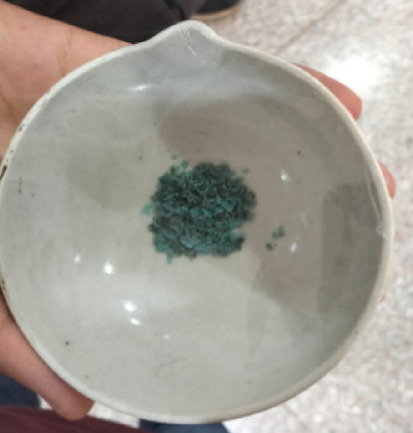
Photograph of synthesized Cu-CDs/TiO_2_.

To prepare the metal composite, mix 3% CuSO_4_ with 50 ml of TiO_2_/CDs. Add to the mixture 50% nitric acid (HNO_3 (aq)_) to adjust the pH to 3.2, then sonication was continued for 2 h. The mixture was stored at room temperature for about a day to allow the powder to settle and then dehydrated in an oven at 85 °C for 12 h. This powder was then transferred into a porcelain dish and kept in a muffle furnace at 500 °C for 2 h^15^. Figure 4 shows the formation of the Cu-CDs/TiO_2_ composite.

### Photocatalytic reduction of CO_2_

**Fig. 5:**
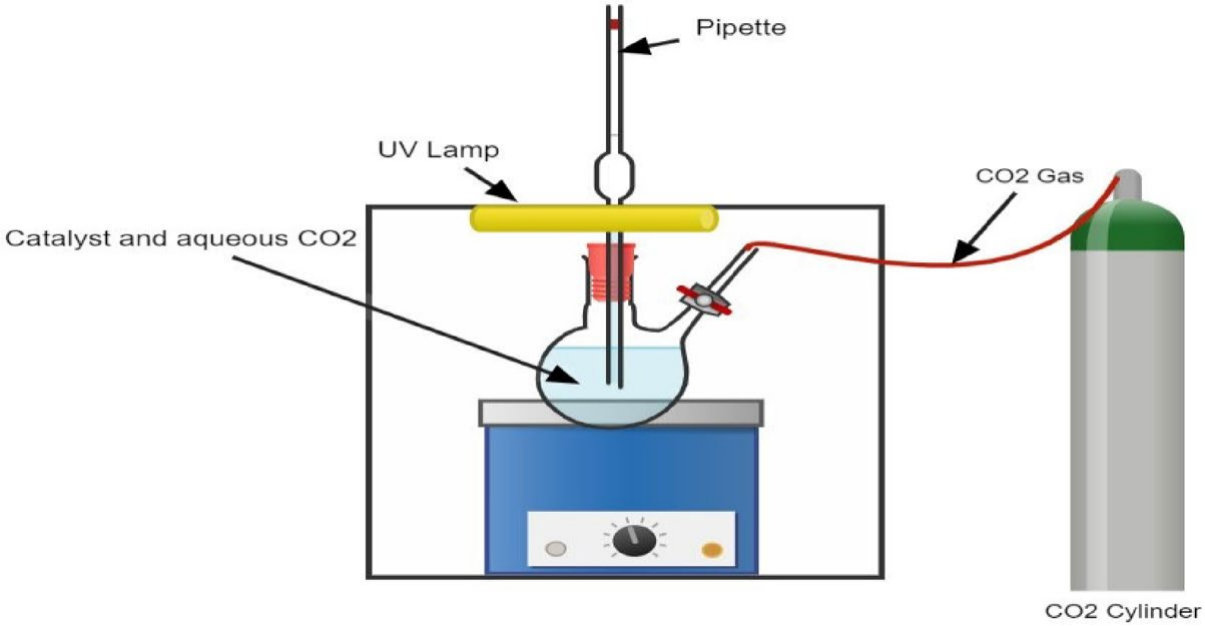
Schematic representation of the batch reactor.

A batch reactor was employed for the photocatalytic reduction tests. The reactor consisted of a three-neck Pyrex glass bottle connected to a CO_2_ cylinder through a securely sealed tube. All the systems were encased in brown glass to shield against UV rays. Figure 5 illustrates a basic schematic depiction of the reaction system. The selected catalyst was introduced into the reactor, and to prevent catalyst sedimentation, all photocatalytic studies were conducted with constant stirring. Before UV-C lamp irradiation, the agitated suspension was purged with CO_2_ for 45 min to achieve a saturated solution, after which the CO_2_ cylinder was deactivated. The photoreactor was securely sealed to ensure consistent CO_2_ pressure and prevent external interaction. The temperature and pH were consistently monitored with a thermometer within the reactor during the reaction. The reactor vessel was subjected to a UVC lamp emitting at a wavelength of 254 nm and a power of 20 W (Sankyo Denki, Germicidal Lamp, G13 T8-Japan). Aqueous samples were extracted at regular intervals using a sealed pipette linked to the reactor through an auxiliary aperture. Samples were collected from the solution at 30-minute intervals. Control experiments were conducted under UV irradiation without a catalyst. CO_2_ reduction levels were assessed via gas chromatography methods.

The materials were examined utilizing an Agilent 5973 mass selective detector, an Agilent 6890 gas chromatography system, an Agilent 7694 headspace sampler, and an HP 5MS column (25 m x 0.25 mm x 0.25 μm). The carrier gas helium flowrate was established at 1 ml/min. The injector port and detector temperatures were established at 250 °C, with a split mode injection ratio of 50:1. In the analysis, the oven temperature was regulated by a temperature ramp protocol that commenced at 37 °C for 2 min, thereafter rose to 40 °C at a rate of 2 °C. min^− 1^, and sustained for 3 min^15^.

### Characterization

The characterization of nanomaterials was accomplished utilizing several pieces of equipment. The functional groups of the nanomaterial samples were analyzed using Fourier Transform Infrared Spectroscopy (FTIR) (D8 ADVANCE, Bruker, Germany), within the range of 500 to 4000 cm^-1^, employing 32 scans and a resolution of 4 cm^-1^. The morphology and structure of CDs were analyzed by Transmission Electron Microscopy (TEM) pictures at a voltage of 200 kV. The diameter of the CDs was determined from the TEM image acquired using a Zeiss-EM10C-100 kV transmission electron microscope (Carl Zeiss, Germany). Image analysis was performed using Image-Pro Plus 6.0 software.

## Result and discussion

### Fourier-transform infrared spectroscopy (FTIR)

**Fig. 6:**
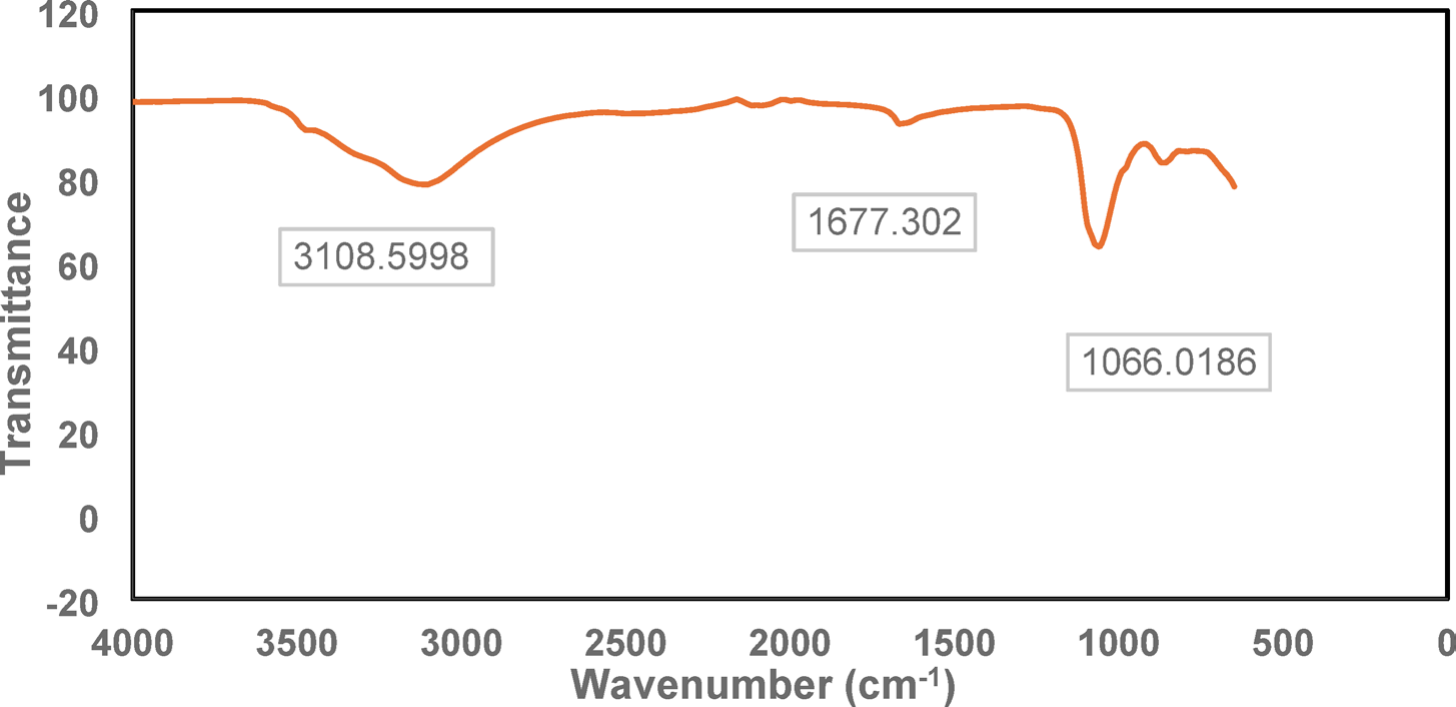
FTIR of the CDs sample.

**Fig. 7:**
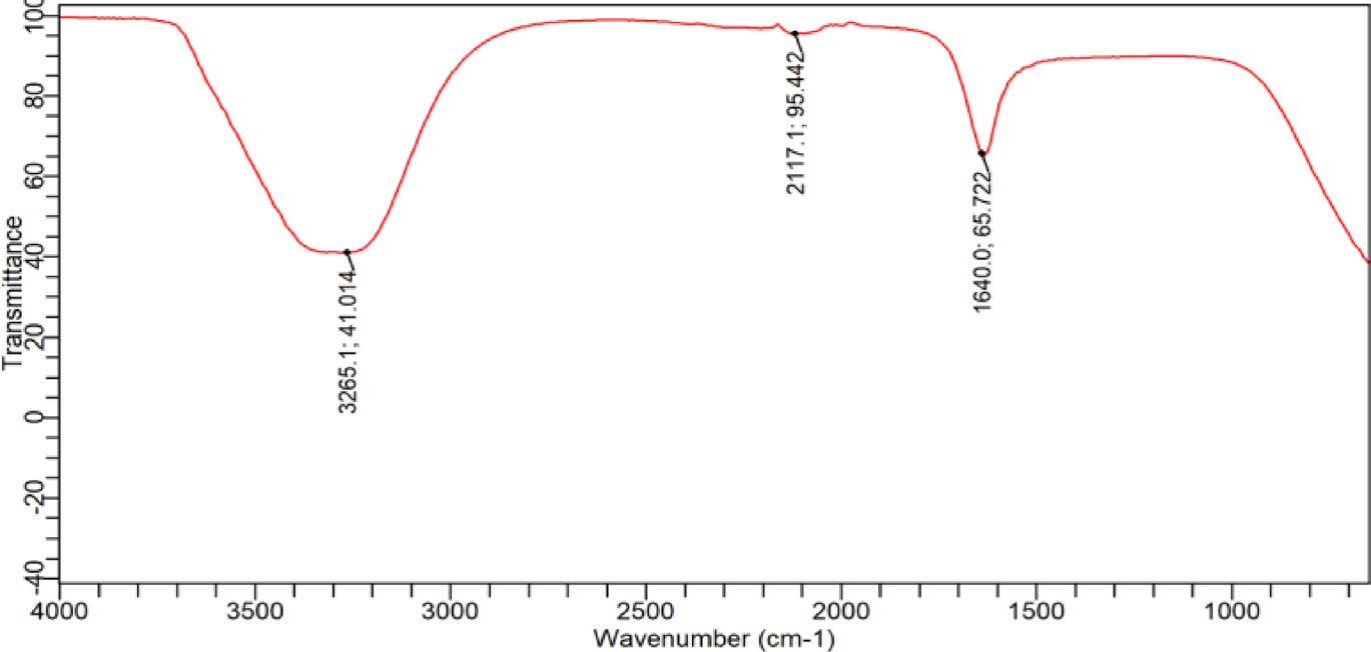
FTIR of the TiO_2_-CDs sample.

FTIR measurements were used to investigate the interaction between TiO₂ and CDs. Figures 6 and 7 illustrate the functional groups of the TiO₂–CDs composite and CDs, respectively. As shown in Fig. 6, the band at 1677.3 cm⁻¹ is attributed to C = O and COO⁻ groups present on the surface of CDs, while the peak at 1066 cm⁻¹ is a characteristic band associated with the formation of carbon-based nanoparticles. In addition, the peak at 3108.6 cm⁻¹ corresponds to O–H stretching vibrations. For the TiO₂–CDs composite Fig. 7, this O–H stretching band shifts to a higher wavenumber 3265.1 cm⁻¹, indicating an interaction between CDs and TiO₂. This shift suggests that CDs contribute to the formation of the composite by providing binding sites, supporting the occurrence of physical adsorption rather than chemical bonding^16^.

### Transmission electron microscopy (TEM)

**Fig. 8:**
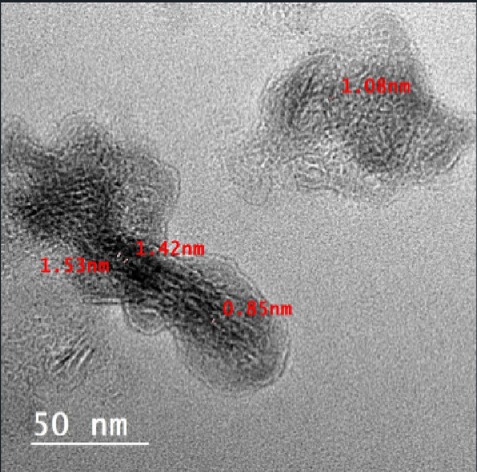
TEM Images of CDs composites.

TEM was employed to acquire nanometer-scale pictures of CDs. Figure 8 illustrates bright-field transmission electron microscopy images of the CDs samples. Spherical nanoparticles measuring between 0.85 and 1.50 nm were detected. Literature has documented TEM pictures of CDs with particle sizes above 0.85 nm, with agglomeration frequently cited as the primary reason. Due to their diminutive size, CDs typically agglomerate in solid form. All samples exhibited nanoparticle agglomeration; furthermore, aggregation may have resulted from the drying process during sample preparation for TEM investigation^17,18^.

### Measurement of the Dissolved CO_2_ in Water

**Fig. 9:**
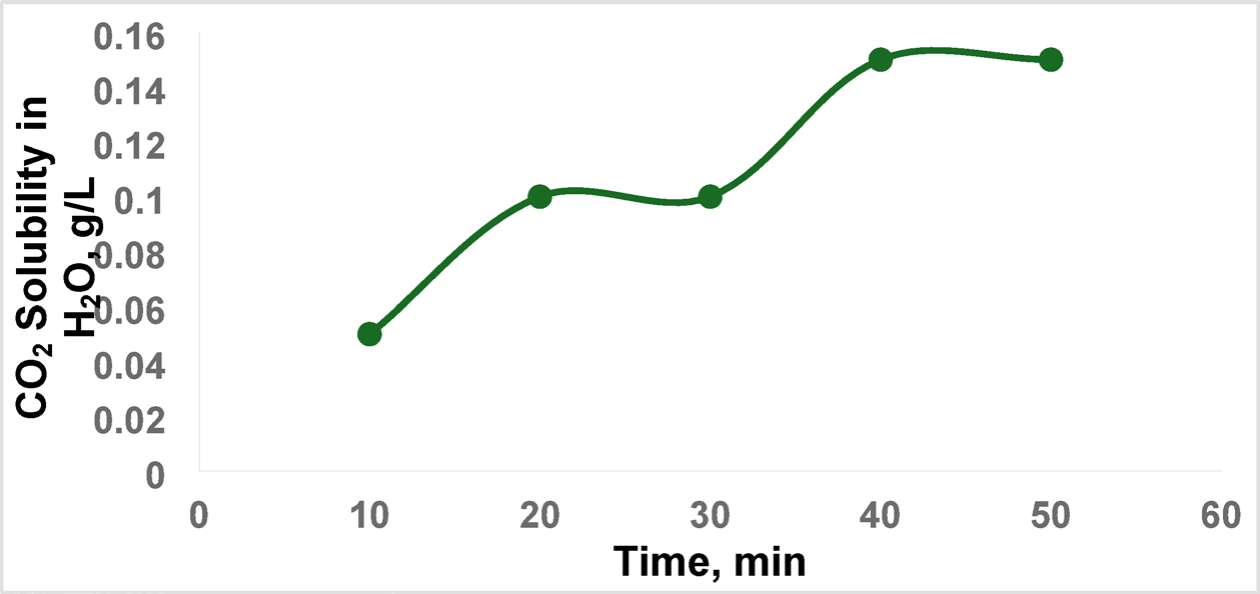
Measurement of the dissolved CO_2_ in water.

Several experiments were conducted to determine the time required to dissolve the maximum amount of CO_2_ in water. The CO_2_ air hose was connected to the flask, and it was allowed to bubble for a set amount of time. The optimum time for maximum CO_2_ dissolution in water was determined.

by titrating 10 ml with 0.1 M NaOH_(aq)_ solution and phenolphthalein, as shown in Fig. 9. The solubility of CO_2_ in water increased over time and reached equilibrium at 40 min.

### Effect of pH on the solubility of CO_2_

**Fig. 10:**
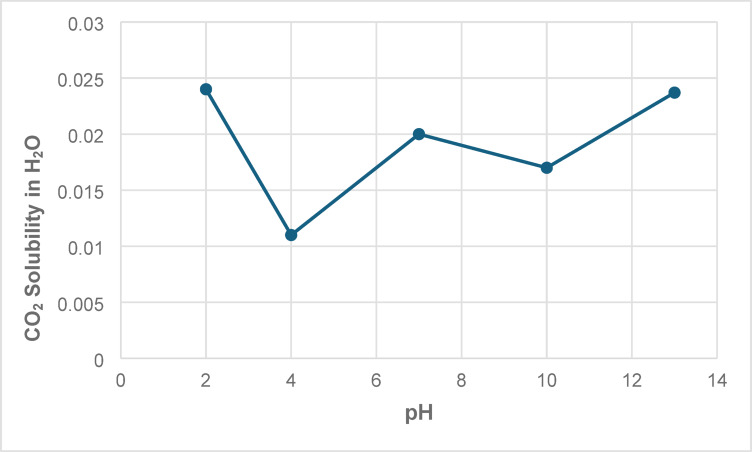
The effect of pH on the solubility of CO_2_.

The pH of the solvent plays a critical role in semiconductor-based photocatalytic reactions, as it influences both the theoretical reduction potential of CO₂ and its aqueous speciation by altering proton concentration. As shown in Fig. 10, the solubility of CO₂ is generally higher under acidic conditions due to increased formation of dissolved CO₂ and carbonic acid (H₂CO₃), while under alkaline conditions, CO₂ is converted into bicarbonate (HCO₃⁻) and carbonate (CO₃²⁻) species. At low pH, dissolved CO₂ and H₂CO₃ are the dominant species due to the high concentration of H⁺ ions. While at a neutral pH (equal to 7), bicarbonate (HCO₃⁻) is the predominant species. At higher pH values (above 10), carbonate ions (CO₃²⁻) dominate the system. However, it should be noted that dissolved CO₂ does not completely disappear above pH 7; rather, its concentration decreases significantly as it is converted into bicarbonate (HCO₃⁻) and carbonate (CO₃²⁻) species^19,20^.

### Effect of acid and alkaline medium on the performance of nano TiO_2_

**Fig. 11:**
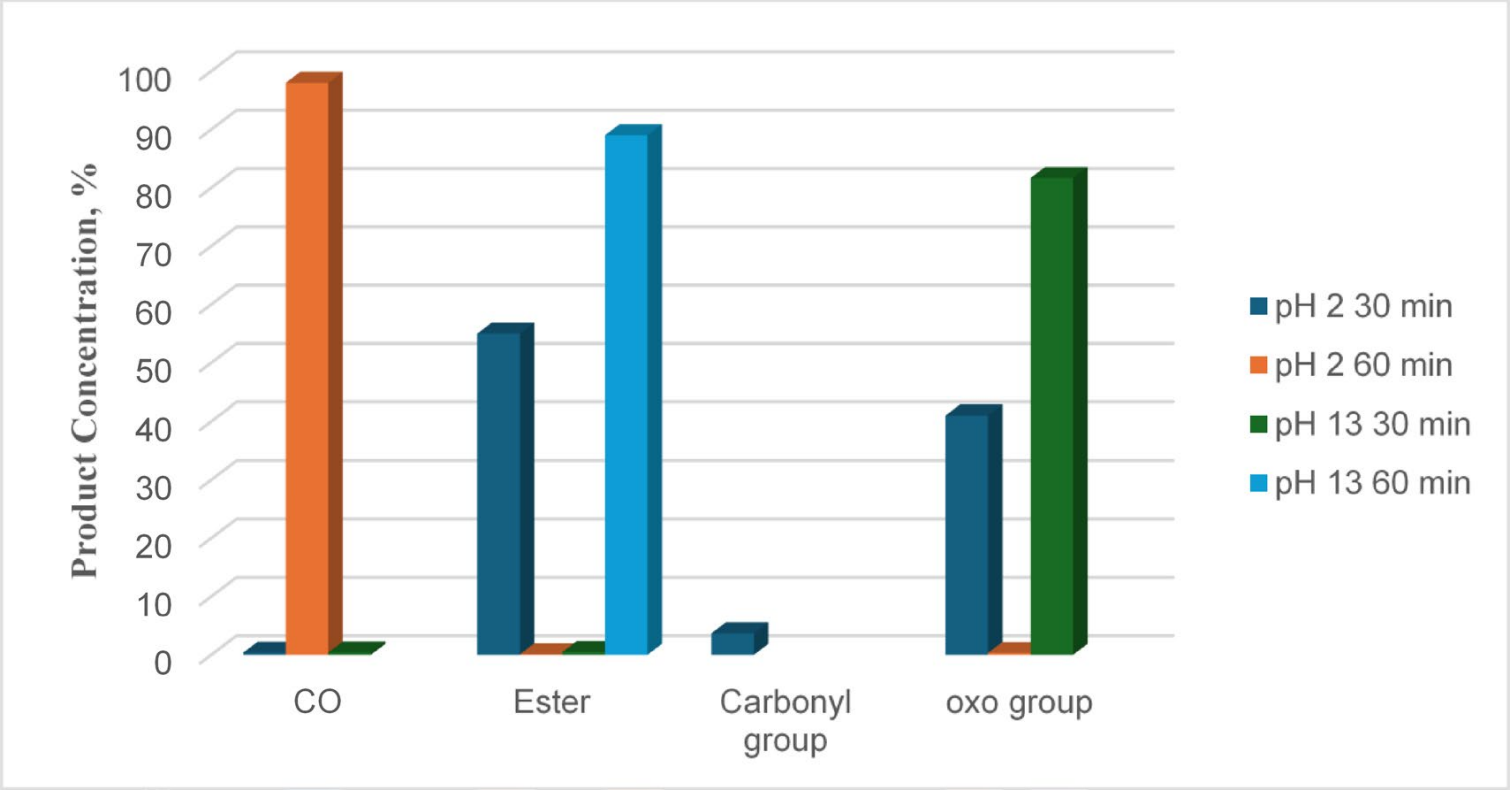
Effect of photocatalytic reduction of CO_2_ using TiO_2_.

The photocatalytic reduction of CO_2_ in an alkaline environment (pH equal 13) yields 81.68% of oxo group at 30 min and 89% esters at 60 min. The oxygenated compounds (oxo groups) become dominant at intermediate times (60–90 min), confirming the formation of intermediate species such as carbonyls and carboxylic acids. This suggests that high pH promotes CO₂ conversion through bicarbonate and carbonate intermediates, which favor oxygenated product formation such as esters. While at pH equal to 2, 55% of ester and 44% of oxo group were produced at 30 min, indicating an early-stage surface intermediate as indicated in Fig. 11. However, after 60 min, CO becomes the major product of 98%, which may indicate reverse reactions or intermediate breakdown. In photocatalytic reactions under high light intensity at 245 nm, the creation of percarbonates as a side reaction resulted solely in the release of OH^−^, leading to the rapid conversion into the carboxylate ester complex from a tetracarbonyl species^21^. Simultaneously, the photocatalytic reduction of CO_2_ in an acidic environment yields 98% CO, 55% esters, and 41% methoxy groups. As the volume of water escalates, a condensed water layer develops on the catalyst surface. The acidic characteristics of the catalyst interface evolve during the reaction, altering the reaction mechanism and promoting the direct dissociation of formate to CO, and to a lesser degree, to methoxy species in the initial 30 min of reduction^22^. Overall, the results demonstrate that alkaline conditions favor esterification pathways, whereas acidic conditions promote a transition from methoxy intermediates toward CO-dominated products with increasing reaction time.

### Performance of carbon dots on the reduction of CO_2_

**Fig. 12:**
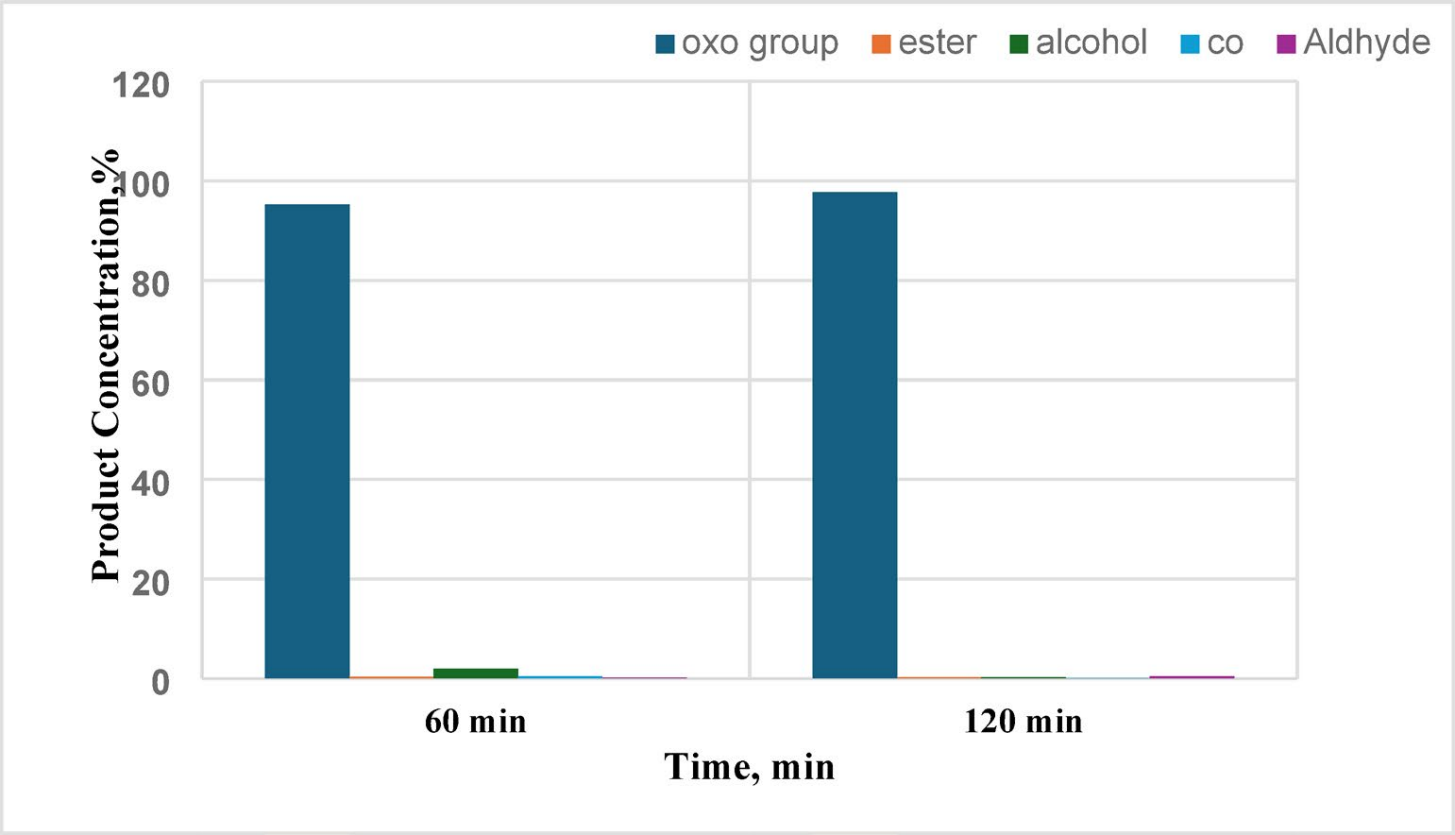
The photocatalytic product using 50 ml of CDs as a photocatalyst.

CDs are synthesized through the hydrothermal decomposition of agricultural waste, yielding CDs with an amorphous carbon core and surface functional groups^23^. Figure 12 illustrates the photocatalytic products produced from the reduction of CO_2_ in an aqueous solution with a CDs photocatalyst. The photocatalytic reaction comprises two half-reactions: the oxidation of water and the multi-electron reduction of CO_2_. Photocatalysis is employed in reduction processes to generate C_1_ products (formic acid, carbon monoxide, formaldehyde, carbon, methanol, and methane) and C_2_ products (oxalic acid, acetic acid, acetaldehyde, ethanol, ethylene, and ethane)^24^. The results shown in Fig. 12 are that oxo group products reach approximately 95.22% at 60 min and 97.73% at 120 min. However, alcohol concentration decreases from 1.97% to 0.29% over time. The concentration of CO and ester is negligible, < 1%. This trend indicates that the reaction favors further oxidation toward oxo products with increasing reaction time. The low concentrations of CO and alcohol suggest that the reaction likely proceeds via the glyoxal pathway.

**Fig. 13:**
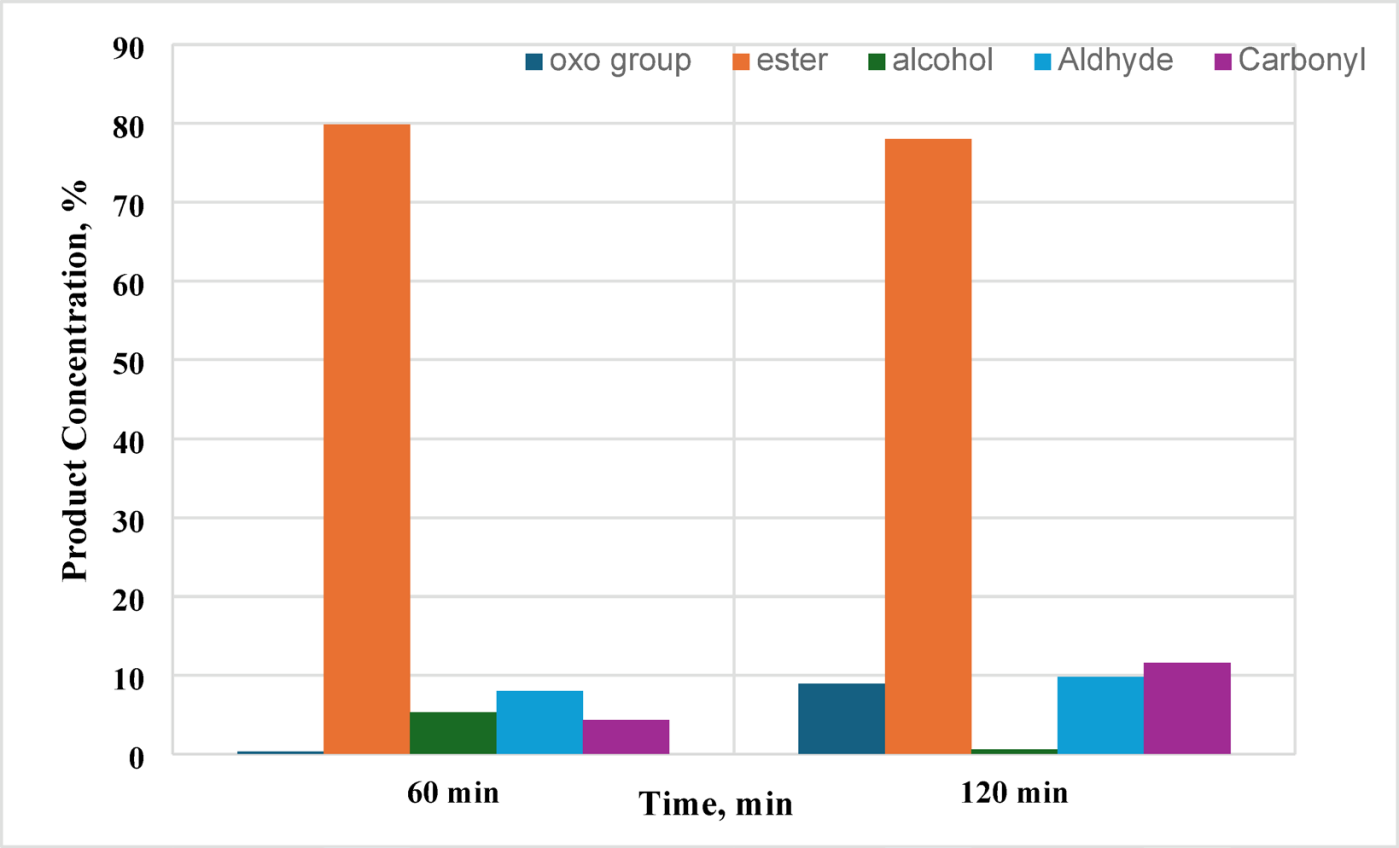
The photocatalytic product using 100 ml of CDs as a photocatalyst.

Furthermore, increasing the amount of CDs to 100 mL, as shown in Fig. 13, increases the number of active sites on the catalyst surface, resulting in a fourfold increase in the alcohol percentage in the solution to 5.3%. In comparison, the esters reached 80% by diminishing the oxygen content to around 0.31%. This behavior indicates that CDs can promote electron transport and prevent charge recombination, hence enhancing the catalytic reaction kinetics of CO_2_ reduction^25–27^.

### Effect of Composite of CDs/TiO_2_

**Fig. 14:**
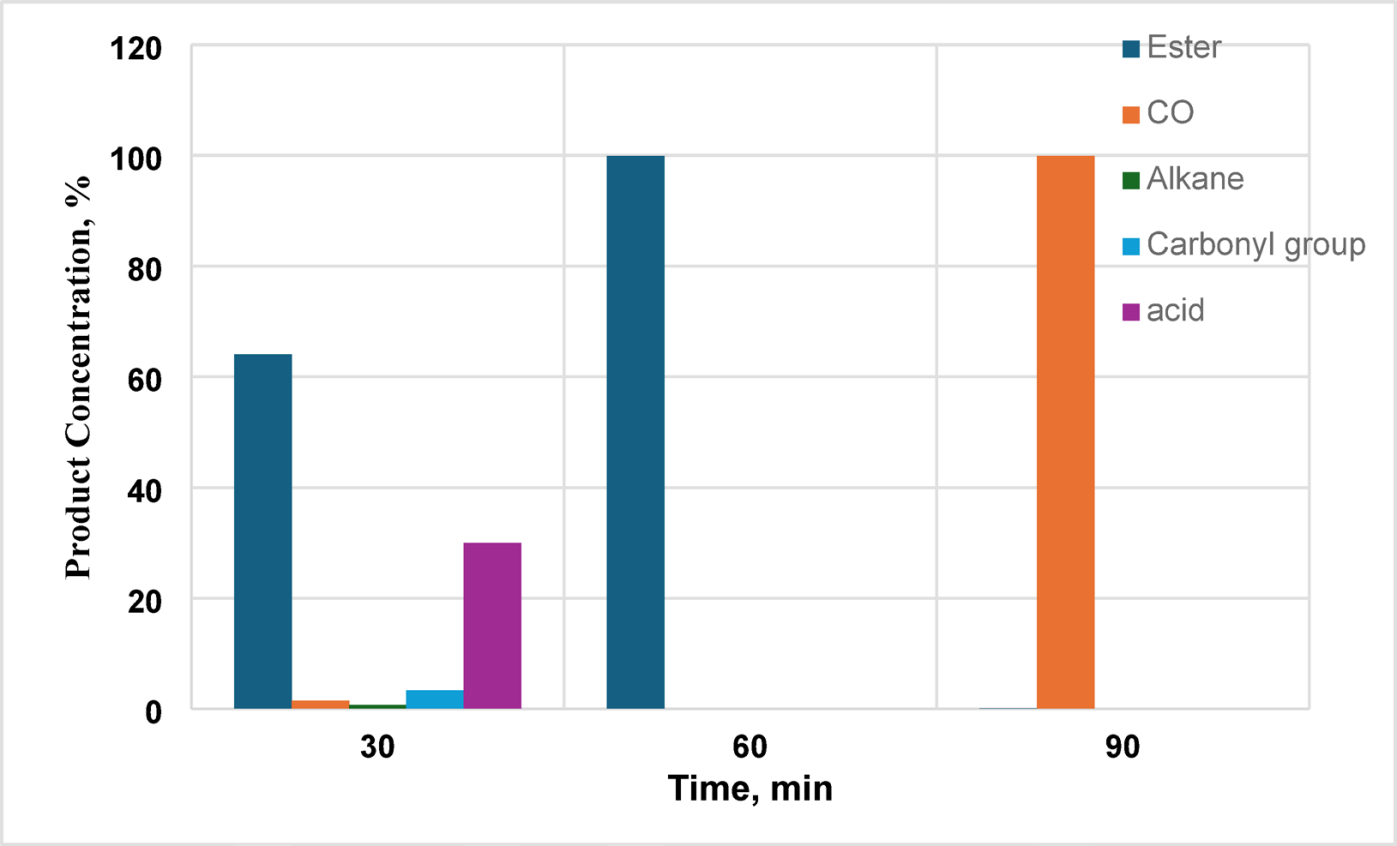
The photocatalytic product using CDs /TiO_2_ as a photocatalyst.

The electron transfer mechanism can explain the photocatalytic system of TiO_2_/CDs used to reduce CO. The UV light irradiation (245 nm), CDs act as photosensitizers. There are two conversion passes. First, the electrons of the conjugated CDs are excited from the valence band to the conduction band, which begins with photon absorption. The photogenerated electrons are then transferred to TiO_2_’s conduction band levels, resulting in the photocatalytic reduction of CO_2_. The electron donor TEOS also serves as a hole scavenger, supplying the system with regeneration energy. The photocatalytic reduction of CO_2_ using TiO_2_/CDs as a photocatalyst promotes the formation of CO. The 30% carboxylic acid complexes shown in Fig. 14 were assumed to be intermediates and used as oxidizers.

The inclusion of 5% TEOS in the composite solution enhances the durability, efficiency, and selectivity of photocatalytic CO_2_ reduction to CO. TEOS facilitates CO_2_ capture, and during the early phase of the photocatalytic reaction, a carbonate ester complex is generated, catalyzing CO_2_ reduction. These photocatalytic systems function as two-component systems, comprising the carbonate ester complex as a catalyst and the composite, which possesses an excited state that may be reductively quenched by TEOS, serving as a photosensitizer^28^.

CDs function as the locus for active hydrogen (H^•^) creation by sequestering H^+^ and e^−^. Therefore, facilitating the activation of both reaction pathways. Two conversion procedures were employed for the acid complex (COOH) intermediate. The primary process, particularly at elevated light intensity, involved the reduction of acid (COOH) by photoinduced electron transfer, culminating in the liberation of 1.97% CO. In a side reaction, acid (COOH) exclusively released OH^−^, resulting in the formation of a tetracarbonyl species at 3.35%, which was rapidly transformed into the carboxylate ester complex through the nucleophilic attack of TEOS. The complex ester (CO- TEOS) possesses a somewhat extended lifespan; nonetheless, its reduction results in the liberation of CO from this intermediate after 90 min, achieving 99.87% yield^29^.

### Effect of metal composite on the photocatalytic reduction of CO_2_

**Fig. 15:**
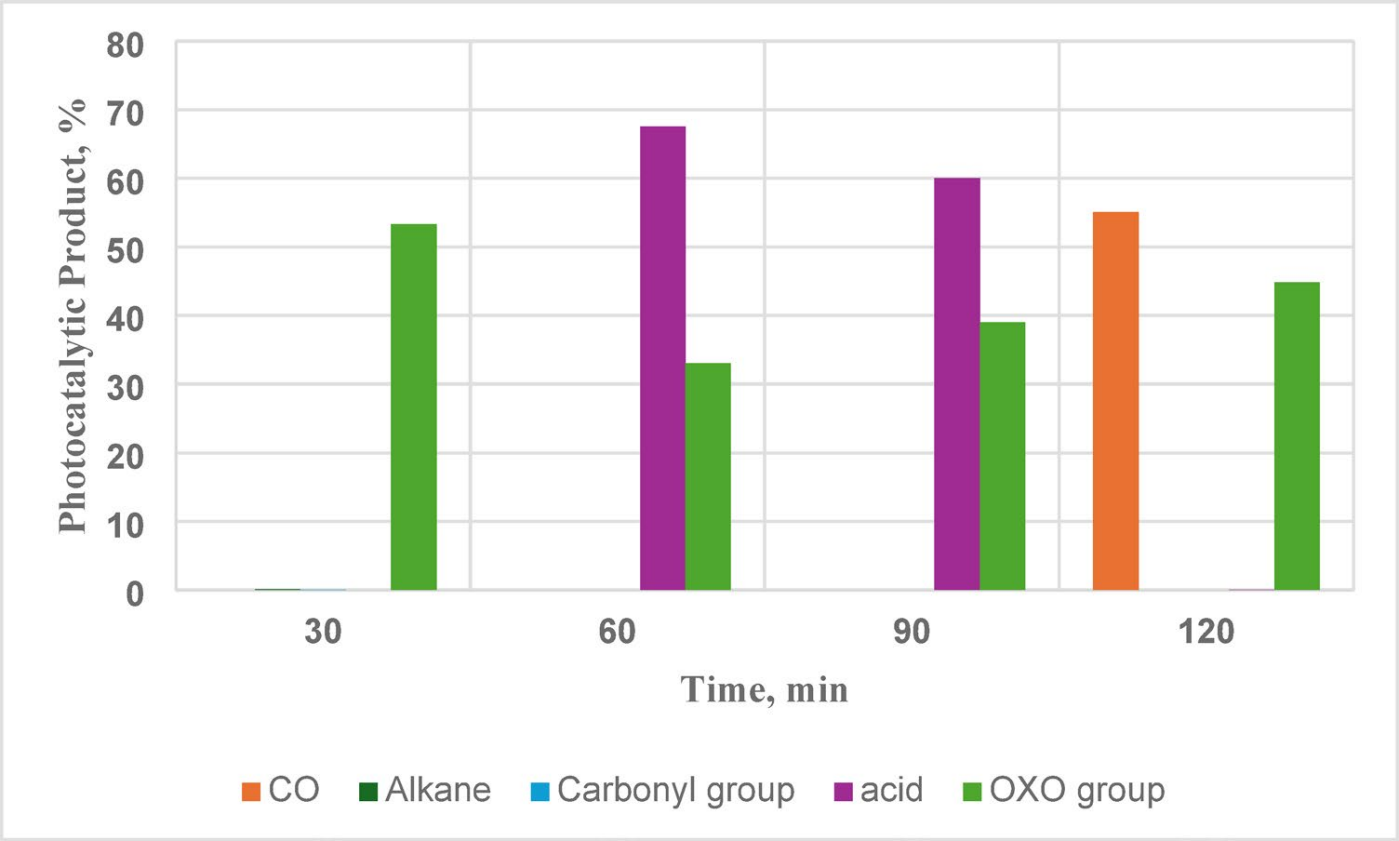
The effect of photocatalytic reduction of CO_2_ using a metal composite.

Figure 15 illustrates the hypothesized pathway for the photocatalytic reduction of CO_2_. Water and CO_2_ molecules interact with confined electrons on the Cu-CDs/TiO_2_ catalyst surface^15^. Cu sites facilitate the electron accumulation and activation of the reactants. The presence of NaOH facilitates the dissociation and transformation of CO_2_ into bicarbonate (HCO₃⁻) and carbonate (CO₃²⁻) ions. Upon exposure to light, photo-excited electrons interact with carbonate species, yielding 53.27% oxo radicals (intermediates) in the presence of catalysts^30^. Further electron transfer results in the formation of formate ion (HCOO⁻), which is then protonated to yield formic acid (HCOOH) as the primary product at around 63%. The addition of 5% TEOS as an electron-donating agent enhanced electron transfer efficiency and promoted further reduction pathways, including the formation of CO. However, the observed decrease in CO to 55% after 2 h suggests the role as an intermediate rather than a final product. Copper plays a crucial role by facilitating H_2_O or H_2_ dissociation into H adatoms owing to its minimal H_2_ dissociation barrier. These hydrogen species accumulate on the Cu surface and participate in hydrogenation reactions. The feeble H-Cu bonds enable effective hydrogenation of intermediates, leading to the formation of an alkane while limiting the accumulation of CO^31^.

## Conclusion

This research aimed to investigate the possibility of photoreduction of CO2 to valuable products using a natural photocatalyst produced from agricultural waste. The photocatalytic performance of TiO2, CDs, CDs/TiO2, and Cu-CDs/TiO2 was evaluated.

The results indicated that CDs alone showed a higher selectivity toward oxygenated products, reaching up to 97.73%, with minimal formation of CO and alcohols. Increasing the concentration of CDs improves electron transfer and increases active sites, which promotes the formation of alcohols and esters. Furthermore, TiO₂ exhibited a dependence on the pH, where ester formation in alkaline media and CO production occurred in acidic media, indicating the influence of surface chemistry on reaction selectivity. The TiO₂/CDs composite performs a higher photocatalytic efficiency due to enhanced charge separation and electron transfer. The addition of TEOS further improved performance by acting as a hole scavenger and facilitating CO₂ capture, enabling a stepwise reaction pathway from intermediates to ester formation and reaching nearly 100% CO selectivity.

In contrast, the metal composite TiO₂/CDs catalyst changed the reaction pathway by promoting hydrogenation reactions. Copper sites enhanced the electron separation and favored the dissociation of H₂O or H₂ into active hydrogen species (H•), which participated in the hydrogenation of intermediates and favored the formation of alkane. Overall, the study demonstrates the pathway of each catalyst. Although TiO₂/CDs is highly effective for selective CO production, the incorporation of Cu as (Cu-CDs/TiO2 ) introduces hydrogenation functionality, enabling the formation of more reduced hydrocarbons.

Future studies should investigate the photocatalytic reduction of CO_2_ at different temperatures to evaluate the effect of temperature on the fluctuation of the products. In addition, examine different concentrations of the catalyst to determine the optimum catalyst load for maximum yield. Moreover, detailed photoreduction kinetics studies should be conducted.

## Data Availability

The datasets used and/or analysed during the current study are available from the corresponding author on reasonable request.
